# The Combination of Natural Molecules Naringenin, Hesperetin, Curcumin, Polydatin and Quercetin Synergistically Decreases SEMA3E Expression Levels and DPPIV Activity in In Vitro Models of Insulin Resistance

**DOI:** 10.3390/ijms24098071

**Published:** 2023-04-29

**Authors:** Emanuele-Salvatore Scarpa, Chiara Giordani, Antonella Antonelli, Massimiliano Petrelli, Giancarlo Balercia, Francesca Silvetti, Alessio Pieroni, Jacopo Sabbatinelli, Maria Rita Rippo, Fabiola Olivieri, Giulia Matacchione

**Affiliations:** 1 Department of Biomolecular Sciences, University of Urbino Carlo Bo, 61029 Urbino, Italy; antonella.antonelli@uniurb.it; 2 Department of Clinical and Molecular Sciences (DISCLIMO), Università Politecnica delle Marche, 60126 Ancona, Italy; c.giordani@pm.univpm.it (C.G.); j.sabbatinelli@staff.univpm.it (J.S.); m.r.rippo@staff.univpm.it (M.R.R.); f.olivieri@staff.univpm.it (F.O.); g.matacchione@pm.univpm.it (G.M.); 3 Clinic of Endocrinology and Metabolic Diseases, Department of Clinical and Molecular Sciences, Università Politecnica delle Marche, 60126 Ancona, Italy; massimiliano.petrelli@ospedaliriuniti.marche.it (M.P.); francesca.silvetti@ospedaliriuniti.marche.it (F.S.); alessio.pieroni2013@libero.it (A.P.); 4 Division of Endocrinology, Department of Clinical and Molecular Sciences, Università Politecnica delle Marche, 60126 Ancona, Italy; Giancarlo.Balercia@ospedaliriuniti.marche.it; 5 Laboratory Medicine Unit, Azienda Ospedaliero Universitaria delle Marche, 60126 Ancona, Italy; 6 Clinic of Laboratory and Precision Medicine, IRCCS Istituto Nazionale di Ricovero e Cura per Anziani, 60121 Ancona, Italy

**Keywords:** natural molecules, insulin resistance, INSR, SEMA3E, caspase 1, DPPIV

## Abstract

Type 2 diabetes mellitus (T2DM) is a disease characterized by a prolonged hyperglycemic condition caused by insulin resistance mechanisms in muscle and liver, reduced insulin production by pancreatic β cells, and a chronic inflammatory state with increased levels of the pro-inflammatory marker semaphorin 3E. Phytochemicals present in several foods have been used to complement oral hypoglycemic drugs for the management of T2DM. Notably, dipeptidyl peptidase IV (DPPIV) inhibitors have demonstrated efficacy in the treatment of T2DM. Our study aimed to investigate, in in vitro models of insulin resistance, the ability of the flavanones naringenin and hesperetin, used alone and in combination with the anti-inflammatory natural molecules curcumin, polydatin, and quercetin, to counteract the insulin resistance and pro-inflammatory molecular mechanisms that are involved in T2DM development. Our results show for the first time that the combination of naringenin, hesperetin, curcumin, polydatin, and quercetin (that mirror the nutraceutical formulation GliceFen^®^, Mivell, Italy) synergistically decreases expression levels of the pro-inflammatory gene *SEMA3E* in insulin-resistant HepG2 cells and synergistically decreases DPPIV activity in insulin-resistant Hep3B cells, indicating that the combination of these five phytochemicals is able to inhibit pro-inflammatory and insulin resistance molecular mechanisms and could represent an effective innovative complementary approach to T2DM pharmacological treatment.

## 1. Introduction

Type 2 diabetes mellitus (T2DM) is a chronic disease characterized by a prolonged hyperglycemic state caused by a combination of underlying defects, which include insulin tolerance in muscle and liver and reduced insulin production by pancreatic β cells. The β cell dysfunction results from deficient glucose sensing and, thus, decreased insulin release, leading to an increase in glucose concentrations in the patients’ blood [1]. Noteworthy genetic and environmental factors, such as a high-fat diet and sedentary lifestyle, can fuel chronic inflammation, worsening hyperglycemia and insulin resistance and thus promoting T2DM progression [2]. Notably, phytochemicals have been used as complements to the most commonly prescribed oral hypoglycemic agents (i.e., sulfonylureas, biguanides, troglitazone, α-glucosidase inhibitors, and thiazolidinediones) for the management of T2DM [3]. Furthermore, conventional drug treatments fail to achieve rigorous metabolic control in more than 50% of T2DM cases, necessitating the search for novel antidiabetic agents that mimic or enhance the properties of insulin as well as protect against diabetic complications [4]. Increasing evidence suggests that natural molecules and functional foods could contribute to ameliorating diabetic syndromes via different mechanisms of action, including enhanced insulin secretion and sensitivity, glucose uptake, and inhibition of glucose absorption from the intestine, along with demonstrated anti-inflammatory properties [5]. In this framework, it was shown that the citrus flavonoids naringenin (NAR) and hesperetin (HES) possess antidiabetic potential, exerted through anti-inflammatory and antioxidant activities and the inhibition of insulin resistance mechanisms [6]. Noteworthy, it was demonstrated that the phytochemicals naringin and its aglycone NAR, have potent anti-diabetic effects in nicotinamide/streptozotocin-induced type 2 diabetic rats via their insulin-improving action, which is mediated through the enhancement of insulin receptor (INSR) and glucose transporter type 4 (GLUT4) levels [7]. Furthermore, it was shown that NAR and HES protected pancreatic β cells in both in vitro and in vivo models; interestingly, the inhibition of thioredoxin-interacting protein expression, regulated by histone acetylation, was critical for the protective role of NAR and HES, which inhibited β cell dysfunction in diabetic db/db mice [8]. Curcumin (CUR) is an active molecule present in the rhizome of the *Curcuma longa* plant [9]. The biological and pharmacological effects of CUR include antioxidant, anti-inflammatory, and hypoglycemic effects [10,11,12]. Polydatin (POL) is a glucoside of resveratrol and is the key component of *Polygonum cuspidatum* [13]. POL has hypoglycemic, antioxidant, and anti-inflammatory activities [14,15]. Interestingly, it was shown that POL treatment improved glucose metabolism by decreasing fasting blood glucose and glycosylated hemoglobin levels in an in vivo experimental model [16]. The flavonoid quercetin (QRC), which can be found in several fruits and vegetables, is able to inhibit lipid peroxidation and possesses anti-inflammatory, antioxidant, and antidiabetic properties [17]. Interestingly, it was shown that QRC and coumarin can remarkably inhibit dipeptidyl peptidase IV (DPPIV) catalytic activity [4]. DPPIV inhibitors provide a potent treatment for T2DM by prolonging the activity of glucagon-like peptide 1 (GLP-1), improving insulin secretion, reducing blood glucose levels, and inhibiting the molecular pathways of insulin resistance [18]. Noteworthy, aberrant activation of the nucleotide-binding domain, leucine-rich containing family, and pyrin domain-containing-3 (NLRP3) inflammasome is responsible for the progression of several human diseases, including septic shock, atherosclerosis, Alzheimer’s disease, and T2DM [19]. Notably, the activation of the inflammasome leads to the maturation and activation of the pro-inflammatory enzyme caspase 1 [20]. It is known that the transcription factor p53 plays a pivotal role in the regulation of glucose metabolism and insulin resistance through molecular pathways involved in the development of T2DM [21]. Indeed, p53 can suppress glucose transport and entry into cells by directly repressing the transcription of the genes glucose transporter type 1 (*GLUT1*), glucose transporter type 3 (*GLUT3*), and *GLUT4*, leading to an increase in blood glucose levels [21]. In addition, it represses the promoter of the *INSR* gene, thereby inhibiting glucose uptake and inducing insulin resistance [21]. Oxidative stress and radical oxygen species (ROS) can activate p53, which induces the transcription of the semaphorin 3E (*SEMA3E*) gene, which is involved in the development of both pro-inflammatory processes and insulin resistance mechanisms [22]. Furthermore, it was shown that the transcription factor p63 can increase the expression levels of sirtuin 1 (*SIRT1*), which is able to deacetylate p53, leading to its destabilization and inactivation [23]. This scientific evidence indicates that p53 activation by metabolic stress can promote diabetes progression by regulating several molecular pathways in adipose tissue, the pancreas, and the liver [21]. In our work, we employed two hepatic cell lines: HepG2 and Hep3B. HepG2 cells represent a *TP53* wt cell line [24], while Hep3B cells represent a *TP53*mut cell line, where p53 function is impaired by the *TP53*-Fragile X mental retardation syndrome-related protein 2 (*FXR2*) fusion [25]. In our study, we investigated, in in vitro models of insulin-resistant hepatic cells [26,27], the ability of the flavanones NAR and HES, used alone and in combination with the natural compounds CUR, POL, and QRC, to counteract the insulin resistance and pro-inflammatory molecular mechanisms that are involved in T2DM development. Notably, it was shown that in young and replicative senescent HUVEC cells exposed to a high-glucose condition and treated with CUR, POL, and QRC, used individually or in combination, the phytochemical combination of these three natural molecules exerted synergistic anti-inflammatory and antioxidant activities [28].

## 2. Results and Discussion

### 2.1. Evaluation of HepG2 and Hep3B Cell Viability after NAR, HES, N + H, C + P + Q, MIX Treatments

**Figure 1:**
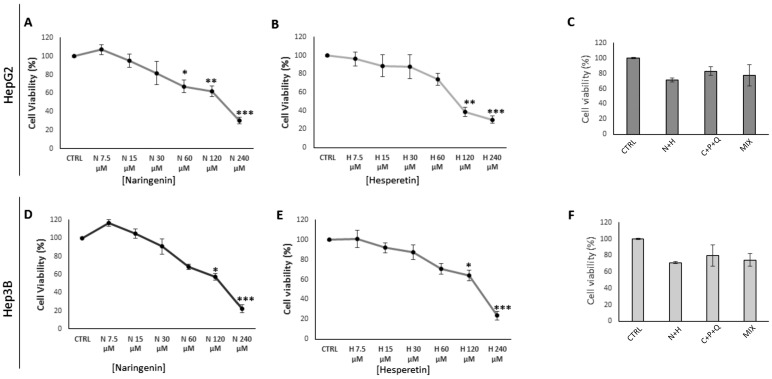
Dose-response curves of HepG2 and Hep3B cell viability after NAR, HES, N + H, C + P + Q, and MIX treatments. HepG2 (**A**–**C**) and Hep3B (**D**–**F**) cells were treated with different concentrations of NAR (0–240 µM), HES (0–240 µM), N + H (30 µM NAR + 30 µM HES), C + P + Q (1 μM CUR + 10 μM POL + 0.5 μM QRC), MIX (N + H + C + P + Q), or 0.4% DMSO alone as a control for 24 h. Results are expressed as a percentage of cell viability compared to the cell viability of 0.4% DMSO-treated cells (CTRL, set to 100%) and presented as mean value ± SD from three independent experiments performed in quadruplicate. * *p* < 0.05, ** *p* < 0.01; *** *p* < 0.001.

We first evaluated the cell viability of HepG2 and Hep3B cells treated for 24 h with different concentrations of NAR and HES, used individually and in combination (N + H) (Figure 1). The results of dose-response cytotoxicity experiments with 0–240 µM NAR and 0–240 µM HES showed that NAR induced a cytotoxic effect in HepG2 cells already at 60 µM concentration with a further decrease of cell viability at 120 µM and 240 µM concentrations (Figure 1A), while in Hep3B cells only the 120 µM and 240 µM NAR treatments induced a significant cytotoxic effect (Figure 1D). When HepG2 and Hep3B cells were treated with the natural compound HES, only the 120 µM and 240 µM HES concentrations were able to significantly decrease the cell viability of both cell lines (Figure 1B,E).

These dose-response experiments allowed us to select the 30 µM treatment of NAR and the 30 µM treatment of HES for the RTqPCR assays, the western blots, the caspase 1 activity assays, and the DPPIV catalytic activity experiments. In addition, we tested the effect of the N + H treatment on the viability of HepG2 and Hep3B cells. Our results showed that the phytochemical combination did not induce any significant cytotoxic effect either in HepG2 (Figure 1C) or in Hep3B (Figure 1F) cell lines, which showed residual cell viability values > 70% (Figure 1). Furthermore, our results indicated that the C + P + Q treatment and the combination of the five natural molecules NAR, HES, CUR, POL, and QRC (MIX) did not induce any cytotoxic effects in both HepG2 and Hep3B cells (Figure 1C,F).

### 2.2. Modulation of INSR, GLUT2, GLUT3, SIRT1, and SEMA3E Gene Expression Levels in HepG2 and Hep3B Cell Lines

**Figure 2:**
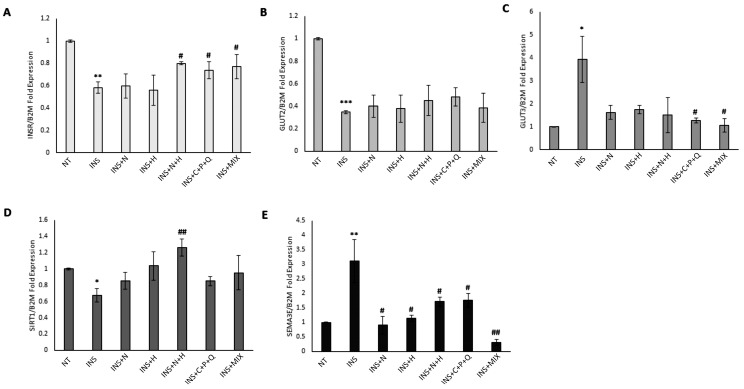
Modulation of *INSR*, *GLUT2*, *GLUT3*, *SIRT1*, and *SEMA3E* expression levels in HepG2 cells. Cells were treated for 72 h with 500 nM insulin (INS) alone or for 72 h with 500 nM INS with the addition of 30 µM NAR, 30 µM HES, N + H (30 µM NAR + 30 µM HES), C + P + Q (1 μM CUR + 10 μM POL + 0.5 μM QRC), and MIX (N + H + C + P + Q) for 24 h. Cells treated with 0.4% DMSO alone for 24 h were used as controls (NT). (**A**) RTqPCR data of the relative expression of *INSR* in HepG2 cells. (**B**) RTqPCR data of the relative expression of *GLUT2* in HepG2 cells. (**C**) RTqPCR data of the relative expression of *GLUT3* in HepG2 cells. (**D**) RTqPCR data of the relative expression of *SIRT1* in HepG2 cells. (**E**) RTqPCR data of the relative expression of *SEMA3E* in HepG2 cells. Results are presented as the mean value ± SD from three independent experiments performed in triplicate. * *p* < 0.05; ** *p* < 0.01; *** *p* < 0.001 when compared with the control (NT) sample; # *p* < 0.05; ## *p* < 0.01 when compared with the INS-treated (INS) sample.

**Figure 3:**
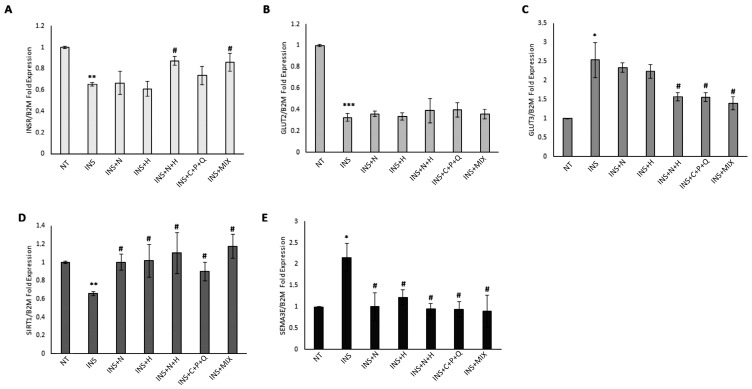
Modulation of *INSR*, *GLUT2*, *GLUT3*, *SIRT1*, and *SEMA3E* expression levels in Hep3B cells. Cells were treated for 72 h with 500 nM insulin (INS) alone or for 72 h with 500 nM INS with the addition of 30 µM NAR, 30 µM HES, N + H (30 µM NAR + 30 µM HES), C + P + Q (1 μM CUR + 10 μM POL + 0.5 μM QRC), and MIX (N + H + C + P + Q) for 24 h. Cells treated with 0.4% DMSO alone for 24 h were used as controls (NT). (**A**) RTqPCR data of the relative expression of *INSR* in Hep3B cells. (**B**) RTqPCR data of the relative expression of *GLUT2* in Hep3B cells. (**C**) RTqPCR data of the relative expression of *GLUT3* in Hep3B cells. (**D**) RTqPCR data of the relative expression of *SIRT1* in Hep3B cells. (**E**) RTqPCR data of the relative expression of *SEMA3E* in Hep3B cells. Results are presented as the mean value ± SD from three independent experiments performed in triplicate. * *p* < 0.05; ** *p* < 0.01; *** *p* < 0.001 when compared with the control (NT) sample; # *p* < 0.05 when compared with the INS-treated (INS) sample.

HepG2 and Hep3B cells were treated for 72 h with 500 nM human recombinant insulin to induce a condition of insulin resistance [26]. Our results show that in both cell lines, there was a significant decrease in *INSR* expression levels (Figure 2A and Figure 3A), indicating that a condition of insulin resistance has been established [26]. RTqPCR data indicate that when NAR and HES were used individually in insulin-treated HepG2 and Hep3B cells, there was not a statistically significant increase in *INSR* mRNA levels, while the N + H treatment significantly increased the expression levels of this gene in both insulin-treated HepG2 and Hep3B cells (Figure 2A and Figure 3A).

Interestingly, the increase in *INSR* expression levels induced by the combination of NAR and HES in HepG2 (+36.9%) and Hep3B (+33.7%) was synergic (Table 1), indicating a synergistic inhibition of the molecular mechanisms of insulin resistance in these cell lines.

In addition, C + P + Q treatment was able to significantly increase the *INSR* expression levels only in HepG2 cells, while MIX treatment induced a significant upregulation of *INSR* mRNA levels in both HepG2 and Hep3B cell lines (Figure 2A and Figure 3A). Since it was demonstrated that the hypoglycemic properties shown by the phytochemical ginsenoside Rk3 were exerted through the increase of GLUT2 levels in an insulin-resistant HepG2 in vitro model [29], we evaluated the modulation of *GLUT2* mRNA levels mediated by our natural compounds. Figure 2B and Figure 3B show that there was a remarkable and significant decrease in *GLUT2* expression levels in both insulin-treated HepG2 and Hep3B cells, but the treatments with NAR, HES, N + H, C + P + Q, and MIX were not able to increase *GLUT2* mRNA levels. As it was shown that the phytochemical corosolic acid can modulate insulin resistance mechanisms in hepatic cells by decreasing the expression levels of *GLUT3* [30], we evaluated the effects of our natural compounds on the modulation of *GLUT3* mRNA levels. The insulin treatment significantly increased the *GLUT3* expression levels in both HepG2 and Hep3B cell lines (Figure 2C and Figure 3C), and the C + P + Q and MIX treatments were associated with a significant decrease of *GLUT3* mRNA levels in these cells (Figure 2C and Figure 3C); in addition, only in Hep3B, the N + H treatment induced a statistically significant decrease of *GLUT3* expression levels (Figure 3C). Interestingly, the decrease of *GLUT3* mRNA levels induced by the combination of NAR and HES in Hep3B (−38.4%) was synergic (Table 1). Noteworthy, Sirtuin 1 is an NAD+-dependent protein deacetylase with a critical function in the regulation of glucose metabolism, insulin resistance, inflammation, and oxidative stress [31]. Sirtuin 1 is also involved in the regulation of insulin secretion from pancreatic β cells, and its catalytic activity protects these cells from inflammation and oxidative stress-mediated tissue damage. In this regard, several sirtuin 1 activators exerted a beneficial impact in reversing T2DM-related complications [31]. Figure 2D and Figure 3D show that the insulin treatment significantly decreased *SIRT1* expression levels in both HepG2 and Hep3B cells, while the N + H treatment was able to remarkably increase *SIRT1* mRNA levels in HepG2 (Figure 2D). Interestingly, the increase in *SIRT1* expression levels induced by the combination of NAR and HES in HepG2 cells (+87.1%) was synergic (Table 1). Moreover, Figure 3D indicates that NAR, HES, N + H, C + P + Q, and MIX treatments were all able to significantly increase *SIRT1* mRNA levels in Hep3B cells. Notably, our RTqPCR results show that there was a statistically significant increase in *SEMA3E* expression levels in the insulin-treated HepG2 and Hep3B cells (Figure 2E and Figure 3E). Noteworthy, NAR, HES, N + H, C + P + Q, and MIX treatments were all able to significantly decrease the *SEMA3E* mRNA levels in both cell lines (Figure 2E and Figure 3E). In particular, the decrease of *SEMA3E* expression levels induced by the MIX treatment in HepG2 cells (−90.1%) was higher than the downregulation mediated by the sum of the effects of the N + H (−45.1%) and C + P + Q treatments (−43.4%), indicating a synergistic inhibition of the molecular mechanisms of insulin resistance and inflammatory conditions exerted by the combination of these five natural molecules in these cells (Table 1). Notably, the values of *SEMA3E* mRNA levels after MIX treatment in HepG2 cells were significantly different when compared with the *SEMA3E* mRNA levels obtained after N + H (*p* = 0.009) and C + P + Q (*p* = 0.005) treatments. We hypothesize that the synergistic and remarkable decrease of *SEMA3E* expression levels exerted by the MIX treatment in only HepG2 cells could be associated with the different *TP53* status of HepG2 (p53 wt) and Hep3B (p53-Null) cell lines since it was shown that p53 induces the transcription of the *SEMA3E* gene [22]. Interestingly, Tsvetkov et al. [32] have demonstrated that CUR inhibits the activity of NADPH quinone oxidoreductase 1, leading to the ubiquitin-independent degradation of p53. In addition, Zeng Z. et al. [33] have shown that POL-dependent activation of the deacetylase sirtuin 1 induced a decrease in the levels of acetylated p53, leading to the inactivation of this transcription factor and a decrease in the expression levels of the genes regulated by p53. This scientific evidence indicates that the synergistic decrease in *SEMA3E* mRNA levels exerted by the MIX treatment in HepG2 cells could be associated with the combined effects of CUR and POL phytochemicals. Notably, it was shown that semaphorin 3E promotes the insulin resistance condition through the inhibition of Ak strain-transforming (AKT) phosphorylation, a marker involved in the activation of insulin signaling [34]. Interestingly, it was demonstrated that patients with diabetes show increased plasma levels of semaphorin 3E, indicating a role for semaphorin 3E-plexin D1 (semaphorin 3E receptor) signaling in diabetes development [22]. In fact, the same authors have shown in an in vivo experimental model that delivery of a soluble form of plexin D1 was able to counteract the detrimental effects of semaphorin 3E, including the recruitment of macrophages in adipose tissue, the macrophage-mediated release of pro-inflammatory molecules Interleukin-6 (IL-6) and tumor necrosis factor-α (TNF-α), and the induction of insulin resistance mechanisms [22].

### 2.3. Modulation of INSR and Semaphorin 3E Protein Levels in HepG2 and Hep3B Cell Lines

**Figure 4:**
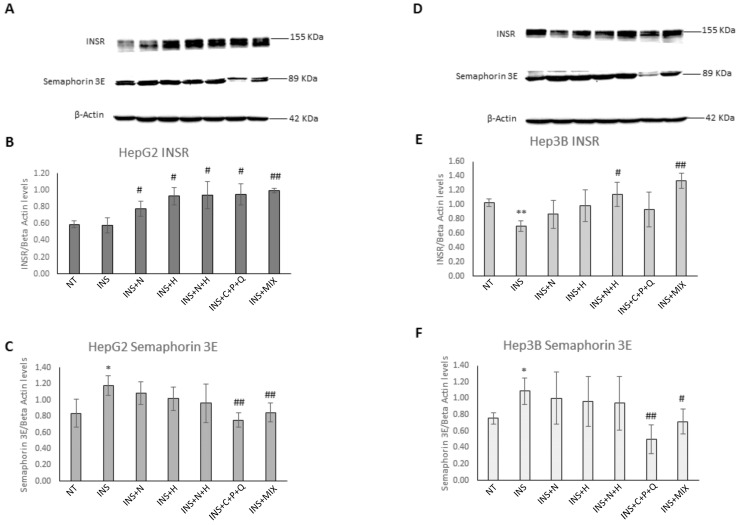
Modulation of INSR and semaphorin 3E protein levels in HepG2 and Hep3B cells. Cells were treated for 72 h with 500 nM insulin (INS) alone or for 72 h with 500 nM INS with the addition of 30 µM NAR, 30 µM HES, N + H (30 µM NAR + 30 µM HES), C + P + Q (1 μM CUR + 10 μM POL + 0.5 μM QRC), and MIX (N + H + C + P + Q) for 24 h. Cells treated with 0.4% DMSO alone for 24 h were used as controls. (**A**) Representative western blot analysis showing INSR and semaphorin 3E protein levels in HepG2 cells not treated (NT) or treated with INS, INS + N, INS + H, INS + N + H, INS + C + P + Q, and INS + MIX; β-actin levels were used as loading controls. (**B**) Graphical representation of western blot data of INSR protein levels, normalized to β-actin protein levels, in HepG2 cells. (**C**) Graphical representation of western blot data of semaphorin 3E protein levels, normalized to β-actin protein levels, in HepG2 cells. (**D**) Representative western blot analysis showing INSR and semaphorin 3E protein levels in Hep3B cells not treated (NT) or treated with INS, INS + N, INS + H, INS + N + H, INS + C + P + Q, and INS + MIX; β-actin levels were used as loading controls. (**E**) Graphical representation of western blot data of INSR protein levels, normalized to β-actin protein levels, in Hep3B cells. (**F**) Graphical representation of western blot data of semaphorin 3E protein levels, normalized to β-actin protein levels, in Hep3B cells. Results are presented as mean value ± SD from three independent experiments. * *p* < 0.05, ** *p* < 0.01 when compared with the not-treated (NT) sample; # *p* < 0.05, ## *p* < 0.01 when compared with the INS sample.

As it was shown that the increase of INSR mRNA and protein levels plays a pivotal role in counteracting insulin resistance mechanisms [35] and that the protein Semaphorin 3E promotes the insulin resistance condition through the inhibition of Akt phosphorylation, a marker involved in the activation of insulin signaling [34], we evaluated, through western blot assays, if NAR, HES, N + H, C + P + Q, and MIX treatments were able to modulate the protein levels of these two markers involved in insulin resistance molecular pathways and in T2DM development. Insulin signaling begins with the binding of this hormone to its cell surface receptor, activation of the receptor tyrosine kinase, and tyrosine phosphorylation of insulin receptor substrate (IRS) proteins, which assemble a signaling complex that includes proteins such as phosphoinositide 3-kinase (PI3K) [35]. This event generates an increase in phosphatidylinositol 3,4,5-trisphosphate levels, leading to the recruitment of Ser/Thr protein kinases such as AKT and 3-phosphoinositide-dependent kinase 1 (PDK1), which phosphorylates AKT at one of its activating sites (Thr308). Partially active AKT phosphorylates and activates mammalian target of rapamycin complex 2 (mTORC2), which phosphorylates AKT at Ser473, leading to full AKT activation [35]. AKT plays a pivotal role in the insulin signaling pathway as it has more than 100 substrates and mediates most, if not all, of the physiological metabolic actions of insulin. Noteworthy, AKT plays an important role in insulin regulated GLUT4 translocation, at least in part by phosphorylating TBC1 domain family member 4 (TBC1D4), which controls the activity of the GTPase RAB10, which induces GLUT4 translocation to the cell membrane, leading to an increase in glucose uptake by the cells [36]. Interestingly, Figure 4A,B shows that in HepG2 cells, all the phytochemical treatments significantly increased the protein levels of INSR, in particular the MIX (Figure 4B), demonstrating an inhibition of the molecular pathways of insulin resistance. Noteworthy, in Hep3B cells, the INS treatment induced a significant decrease in the INSR protein levels (Figure 4D,E). Notably, Figure 4E shows that only the N + H and MIX treatments were able to significantly increase the INSR protein levels, indicating that the combination of these natural molecules can counteract the insulin resistance mechanisms in Hep3B cells. Interestingly, Figure 4A,C,D,F shows that in HepG2 and Hep3B cells, the insulin treatment induced a significant increase in semaphorin 3E protein levels, and only the C + P + Q and MIX treatments were able to significantly decrease the levels of this pro-inflammatory marker (Figure S1 shows the uncropped blots of images of Figure 4). Our results indicate that these natural molecules can counteract both the pro-inflammatory and insulin resistance pathways modulated by semaphorin 3E. Notably, the protein levels of semaphorin 3E after the MIX treatment in Hep3B cells were significantly different when compared with the semaphorin 3E levels obtained after N + H treatment (*p* = 0.038) and were similar to those obtained after C + P + Q treatment (*p* = 0.081). To the best of our knowledge, our study describes for the first time the remarkable decrease of semaphorin 3E protein levels induced by the combination of natural molecules CUR, POL, and QRC in in vitro models of insulin resistance.

### 2.4. Inhibition of the Catalytic Activity of the Pro-Inflammatory Enzyme Caspase 1 in HepG2 and Hep3B Cells

**Figure 5:**
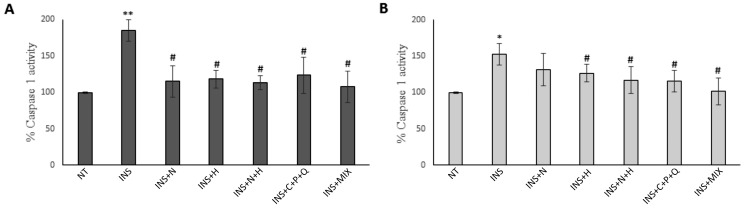
Regulation of caspase 1 catalytic activity in HepG2 and Hep3B cells. Cells were treated for 72 h with 500 nM insulin (INS) alone or for 72 h with 500 nM INS with the addition of 30 µM NAR, 30 µM HES, N + H (30 µM NAR + 30 µM HES), C + P + Q (1 μM CUR + 10 μM POL + 0.5 μM QRC), and MIX (N + H + C + P + Q) for 24 h. Cells treated with 0.4% DMSO alone for 24 h were used as controls (NT). (**A**) Caspase 1 activity levels in HepG2 cells. (**B**) Caspase 1 activity levels in Hep3B cells. Results are presented as mean values ± SD from three independent experiments performed in duplicate. * *p* < 0.05; ** *p* < 0.01 when compared with the not-treated (NT) sample; # *p* < 0.05 when compared with the INS-treated sample.

Noteworthy, it was shown that aberrant activation of the NLRP3 inflammasome is responsible for the progression of several human diseases, including septic shock, atherosclerosis, Alzheimer’s disease, and T2DM [19]. During inflammasome assembly, pro-caspase 1 and sensor proteins are linked by an adapter protein known as an apoptosis-associated speck-like protein containing a caspase-recruitment domain (ASC); this interaction leads to the cleavage and activation of pro-caspase 1 and the maturation of pro-inflammatory cytokines such as Interleukin-1β (IL-1β) or interleukin-18 (IL-18), which are involved in the T2DM pathogenesis [20]. Considering this scientific evidence, we evaluated if NAR, HES, N + H, C + P + Q, and MIX treatments were able to decrease caspase 1 activity in insulin-treated HepG2 and Hep3B cells. Figure 5A,B shows that the insulin treatment significantly increased caspase 1 activity levels in both HepG2 and Hep3B cells, with a higher increase in catalytic activity in the HepG2 cell line. These results indicate that the establishment of the insulin resistance condition induces an inflammation state, which can lead to the development and worsening of the T2DM pathological condition [19]. The NAR, HES, N + H, C + P + Q, and MIX treatments were all able to significantly decrease the caspase 1 activity in HepG2 cells (Figure 5A). In Hep3B cells, the HES, N + H, C + P + Q, and MIX treatments significantly reduced caspase 1 activity levels, while the NAR treatment induced only a slight decrease (Figure 5B).

Interestingly, it was shown that other phytochemicals are also able to inhibit caspase 1 activity: 10-gingerol and the shogaols extracted from ginger [37]. The authors demonstrated that shogaols and 10-gingerol inhibited IL-1β secretion, reduced NLRP3 levels, and decreased the ATP-induced activation of the pro-inflammatory enzyme caspase 1 [37]. Regarding the modulation of inflammasome activity by the investigated phytochemicals NAR, CUR, and POL, it was demonstrated that NAR was able to inhibit the activation of caspase 1 and receptor-interacting protein 2 (RIP2) in the human mast cell line HMC-1 [38]. Furthermore, it was shown that CUR inhibited caspase 1 activation and IL-1β secretion by suppressing the NLRP3-mediated inflammasome activation pathway in an in vitro experimental model [39]. Notably, it was demonstrated that POL was able to decrease caspase 1, ASC, and NLRP3 protein levels in an in vivo experimental model, leading to the inhibition of pro-inflammatory cytokine production in serum and kidney [40].

### 2.5. Inhibition of the Catalytic Activity of the Hyperglycemic Enzyme DPPIV in HepG2 and Hep3B Cells

**Figure 6:**
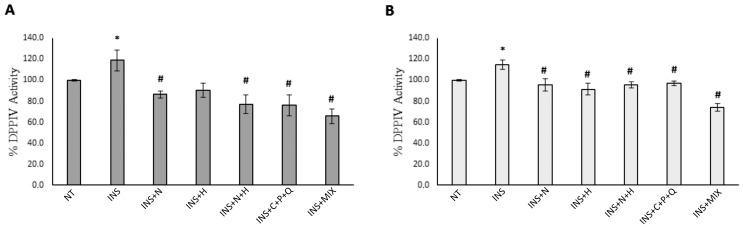
Regulation of DPPIV catalytic activity in HepG2 and Hep3B cells. Cells were treated for 72 h with 500 nM insulin (INS) alone or for 72 h with 500 nM INS with the addition of 30 µM NAR, 30 µM HES, N + H (30 µM NAR + 30 µM HES), C + P + Q (1 μM CUR + 10 μM POL + 0.5 μM QRC), and MIX (N + H + C + P + Q) for 24 h. Cells treated with 0.4% DMSO alone for 24 h were used as controls (NT). (**A**) DPPIV activity levels in HepG2 cells. (**B**) DPPIV activity levels in Hep3B cells. Results are presented as the mean value ± SD from three independent experiments performed in triplicate. * *p* < 0.05 when compared with the not-treated (NT) sample; # *p* < 0.05 when compared with the INS-treated sample.

Interestingly, it was shown that DPPIV inhibitors provide a potent treatment for T2DM, improving insulin secretion, reducing blood glucose levels, and inhibiting the molecular pathways of insulin resistance [18]. Considering this scientific evidence, we evaluated if NAR, HES, N + H, C + P + Q, and MIX treatments were able to decrease the DPPIV activity levels in insulin-treated HepG2 and Hep3B cells. Figure 6A,B indicates that the insulin treatment induced a significant increase in DPPIV activity in both HepG2 and Hep3B cells. Furthermore, Figure 6A shows that the NAR, N + H, C + P + Q, and MIX treatments were able to significantly decrease the DPPIV activity levels in HepG2 cells, while the HES treatment induced only a slight decrease. Figure 6B shows that in Hep3B cells, all the treatments (i.e., NAR, HES, N + H, C + P + Q, and MIX) significantly decreased the catalytic activity of DPPIV. Noteworthy, the decrease of DPPIV activity levels induced by the MIX treatment in Hep3B cells (−35.5%) was higher than the decrease mediated by the sum of the effects of the N + H (−16.3%) and C + P + Q (−15.6%) treatments (Table 1), indicating a synergistic inhibition of the molecular pathways of insulin resistance exerted by the combination of these five natural molecules. Regarding the modulation of the DPPIV enzymatic activity exerted by the investigated phytochemicals NAR, HES, and CUR, it was shown that the natural molecules NAR, cirsimarin, hispidulin (obtained from the plant *Lippia graveolens*), and the bioactives HES, rutin, and eriodictyol, present in three citrus bioflavonoid nutraceuticals, were all able to decrease the catalytic activity of DPPIV [41,42]. Interestingly, it was demonstrated, through molecular docking techniques and in vitro models, that CUR was able to interact with the catalytic site of DPPIV, inhibiting its activity [43]. Notably, it was shown that orally active DPPIV inhibitors, suitable for once-daily administration in patients, have demonstrated efficacy in new therapies for the treatment of T2DM, leading to an increase in GLP-1 levels, a hormone that is cleaved by DPPIV [44]. GLP-1 plays a pivotal role in stimulating insulin secretion, increasing insulin sensitivity, and improving the glycemic profile of patients [44]. Noteworthy, the results of a clinical trial showed that the combination of HES and trans-resveratrol (the aglycone of POL) exerted anti-inflammatory effects and was also able to reverse the insulin resistance condition in overweight and obese patients [45].

## 3. Materials and Methods

### 3.1. Natural Compounds

The standards of the following natural compounds have been used: CUR (A218580100, Carlo Erba, Cornaredo, Italy), POL (P1878, TCI, Haven, Belgium), QRC (P0042, TCI, Haven, Belgium), NAR (N0072, TCI, Haven, Belgium), and HES (H0721, TCI, Haven, Belgium).

### 3.2. Cell Cultures

HepG2 and Hep3B human hepatic cell lines were purchased from the American Type Culture Collection (ATCC, Rockville, Daytona Beach, FL, USA) and maintained in DMEM supplemented with 10% FBS, 2 mM glutamine, 100 U/mL penicillin, 100 μg/mL streptomycin, and 200 μM MEM nonessential amino-acid solution (DMEM complete medium). Cell lines were grown at 37 °C in a humidified atmosphere with 5% CO_2_.

### 3.3. Serum Starvation and Insulin Treatments

HepG2 and Hep3B cells were seeded in 6-wells, 12-wells, or 96-wells, then the cell medium was removed and replaced with DMEM supplemented with 2 mM glutamine, 100 U/mL penicillin, 100 μg/mL streptomycin, and 200 μM MEM nonessential amino-acid solution, but without FBS to obtain serum starvation conditions. After 6 h, this medium was removed and replaced with DMEM complete medium. The treated cells were incubated for 72 h with 500 nM human recombinant insulin to induce insulin resistance, while the control cells were grown in DMEM complete medium. After 48 h of insulin treatment, HepG2 and Hep3B cells were incubated for 24 h with the natural compounds.

### 3.4. Natural Compound Treatments

The natural molecules analyzed in this study are part of the nutraceutical formulation GliceFen^®^ (Mivell, Fano, Italy; Italian Patent Application: 102021000032909). POL, CUR, and QRC were used at concentrations of 10 µM, 1 µM, and 0.5 µM, respectively, as previously described [28]. Based on the results of cell viability assays, HepG2 and Hep3B cells were treated with 30 µM NAR and 30 µM HES. The treatments with the single phytochemicals NAR and HES, the mix of 30 µM NAR and 30 µM HES (N + H), the combination of 1 µM CUR, 10 µM POL, and 0.5 µM QRC (C + P + Q), and the combination of 30 µM NAR, 30 µM HES, 1 µM CUR, 10 µM POL, and 0.5 µM QRC (MIX) were performed for 24 h. Noteworthy, the combination of phytochemicals exerts a synergistic effect when the observed biological effect of the combination of the natural molecules is greater than the sum of the biological effects of the individual natural molecules.

### 3.5. Cell Viability Assay

HepG2 and Hep3B cells were seeded in 96-well plates at a density of 10,000 cells/well and were grown for 24 h. The treated cells were incubated for 24 h with 0–240 µM NAR or 0–240 µM HES or with the combination of 30 µM NAR + 30 µM HES (N + H) or with C + P + Q or MIX treatments. The control cells were incubated for 24 h with 0.4% DMSO. The effect of natural compounds on cell viability was evaluated using the CellTiter 96 AQueous One Solution Cell Proliferation Assay (Promega s.r.l., Milan, Italy). This assay is based on the reduction of the MTS reagent [3-(4,5-dimethylthiazol-2yl)-5-(3-carboxymethoxyphenyl)-2-(4-sulfophenyl)2H-tetrazolium, inner salt] into a colored formazan product that is soluble in culture medium. This conversion is accomplished by NADPH or NADH produced by dehydrogenase enzymes in metabolically active cells. The quantity of formazan product, measured by absorbance at 490 nm with the Biotek Synergy H1 plate reader (Agilent, Santa Clara, California; USA), is directly proportional to the number of living cells in the culture. The results were expressed as a percentage of residual cell viability compared to control cells (CTRL) treated with 0.4% DMSO (set at 100% cell viability) for both HepG2 and Hep3B cell lines.

### 3.6. Real-Time Quantitative Polymerase Chain Reaction (RTqPCR)

HepG2 and Hep3B cells were seeded in 12-well plates at a concentration of 150,000 cells/well and grown for 24 h The cells were then treated as described in Section 3.3 and Section 3.4. In particular, after 48 h of insulin treatment, HepG2 and Hep3B cells were incubated for 24 h with 30 µM NAR or 30 µM HES or with the N + H, C + P + Q, or MIX treatments. For gene-specific expression analysis, total RNA was isolated using the Total RNA Purification Kit of NORGEN Biotek Corp. (Thorold, ON, Canada). A quantity of 1 µg of total RNA was reverse-transcribed using the ImProm-IITM Reverse Transcription System (Promega s.r.l., Milan, Italy) with oligo-dT primers, following the manufacturer’s instructions and according to the following thermal profile: 25 °C for 5 min, 42 °C for 60 min, and 70 °C for 15 min. RTqPCR detection and expression analysis of genes was performed with SYBR green quantitative real-time PCR using the TB green Premix Ex Taq II (Tli RNaseH Plus) Kit (Takara, Kusatsu, Japan), according to the manufacturer’s instructions. Briefly, the reaction was set up in a 20 µL final volume, using 20 ng of cDNA as the template and 200 nM of each specific primer. For RTqPCR amplifications, 40 PCR cycles were run with the following 2-step thermal profile: 5 s at 95 °C and 34 s at 60 °C per cycle; before cycling, 30 s at 95 °C were allowed for Taq DNA polymerase activation. The fluorescence intensity of each amplified sample was measured with the QuantStudio3 sequence detection system (Applied Biosystems, Foster City, CA, USA). All measurements were performed in triplicate and reported as the average values ± standard deviation of the mean (mean ± SD). Target gene values were normalized with β2-microglobulin (B2M) mRNA measurements, and expression data were calculated according to the 2^−ΔΔCt^ Livak method. The sequences of the primers used for RTqPCR assays are reported in Table 2.

### 3.7. Determination of Caspase 1 Activity

HepG2 and Hep3B cells were seeded on 6-well plates at a density of 300,000 cells/well and grown for 24 h. The cells were then treated as described in Section 3.3 and Section 3.4. After 48 h of insulin treatment, HepG2 and Hep3B cells were incubated for 24 h with 30 µM NAR, 30 µM HES, or the N + H, C + P + Q, or MIX treatments. On the day of the experiment, the medium was removed, and the 6-well plates were put on ice. The cells were washed twice with 1 mL of phosphate buffered saline (PBS) 1×, then 100 μL of cell lysis buffer (Enzo Life Sciences, Farmingdale, NY, USA) were added to each well, and the cells were scraped for 2 min. The lysates from each sample were then recovered and transferred into fresh 1.5 mL tubes. The samples were maintained at −80 °C for 30 min, then unfrozen in ice and sonicated for 20 min at room temperature in a water-sonication bath (Ultrasonic Cleaner, BRANSON 200). The samples were centrifuged at 12,000× *g* at 4 °C for 10 min, then the supernatants (the cytosols) were recovered and put into fresh 1.5 mL tubes, as previously reported [46]. The protein concentration of each sample was assessed through the Bradford assay (BioRad Laboratories, Hercules, CA, USA), measuring the absorbance values at λ = 595 nm in a Biotek Synergy H1 plate reader. Caspase 1 activity was assessed through the Caspase Colorimetric Assay Kit (Enzo Life Sciences), as previously reported for other caspases [47]. Briefly, 100 μg of cytosol from untreated or phytochemically treated HepG2 and Hep3B cells were incubated with caspase reaction buffer (25 mM Hepes pH 7.4, 50 mM NaCl, 0.05% Triton, 0.5 mM EDTA, 5% glycerol, 5 mM DTT), then the substrate YVAD-pNA (400 μM and 200 μM final concentrations for HepG2 and Hep3B samples, respectively) specific for caspase 1 was added, and the samples were transferred into a 96-well microplate and were incubated at 37 °C for 2 h. The absorbance, representing the activity of caspase 1, was measured at λ = 405 nm in a Biotek Synergy H1 plate reader.

### 3.8. In Situ Cell-Based DPPIV Activity Assay

HepG2 and Hep3B cells were seeded on black 96-well plates with clear bottoms at a density of 10,000 cells/well and were grown for 24 h. The cells were then treated as described in Section 3.3 and Section 3.4. In particular, after 48 h of insulin treatment, HepG2 and Hep3B cells were incubated for 24 h with 30 µM NAR or 30 µM HES or with the N + H, C + P + Q, or MIX treatments. On the day of the experiment, cells were washed once with 100 µL of PBS, then H-Gly-Pro-AMC (AS-24098, AnaSpec Inc., Fremont, CA, USA) substrate diluted in PBS (5 µM and 1 µM final concentrations for HepG2 and Hep3B cells, respectively) was added, and the fluorescence signals (excitation and emission wavelengths of 353 and 442 nm, respectively) in each well were measured using a Synergy Biotek plate reader after 10 min of incubation, as previously reported [48]. All measurements were performed in triplicate and reported as the average values ± standard deviation of the mean (mean ± SD).

### 3.9. Western Blot Analysis

HepG2 and Hep3B cells were seeded on 6-well plates at a density of 250,000 cells/well and grown for 24 h. The cells were then treated as described in Section 3.3 and Section 3.4. In particular, after 48 h of insulin treatment, HepG2 and Hep3B cells were incubated for 24 h with 30 µM NAR, 30 µM HES, or the N + H, C + P + Q, or MIX treatments. On the day of the experiment, the medium was removed, and the HepG2 and Hep3B cells of the 6-well plates were incubated with 500 μL of trypsin/EDTA 1× solution for 5 min at 5% CO_2_ and 37 °C, then 1 mL of complete DMEM medium was added to each well. The cells were recovered and transferred to sterile 1.5 mL tubes, which were centrifuged at 12,000× *g* for 5 min to obtain the cell pellets that had been stored at −80 °C. Cell lysates were obtained using RIPA buffer (20 mM Tris-HCl pH 7.5; 150 mM NaCl, 1 mM Na_2_EDTA, 1 mM EGTA, 1% NP-40, 1% sodium deoxycholate, 2.5 mM sodium pyrophosphate, 1 mM β-glycerophosphate, 1 mM Na_3_VO_4_, and 1 µg/mL leupeptin) with the addition of protease inhibitor cocktail (Cell Signaling Technology, Danvers, MA, USA). HepG2 and Hep3B samples were sonicated for 20 min at room temperature in a water-sonication bath (Ultrasonic Cleaner, BRAN-SON 200). The samples were centrifuged at 12,000× *g* at 4 °C for 10 min, then the supernatants (the cytosols) were recovered and put into fresh 1.5 mL tubes. The samples were boiled for 5 min and then stored at −80 °C. Protein concentration for each sample was determined by Bradford assay after evaluating the absorbance values at λ = 595 nm. Proteins were resolved by SDS polyacrylamide gel electrophoresis (SDS-PAGE) and electroblotted onto a PVDF membrane (0.2 µm pore size) (Bio-Rad, Hercules, CA, USA). The blots were probed with the following primary antibodies: anti-InsR (#MA1-10865, monoclonal; Thermo Fisher, Waltham, MA, USA), anti-semaphorin 3E (#PA5-75684, polyclonal; Thermo Fisher), and anti-β-actin (#VMA00048, monoclonal; Bio-Rad), which was used as a loading control. Immunoreactive bands were detected by horseradish peroxidase (HRP)-conjugated secondary antibodies (Bio-Rad). Peroxidase activity was detected with the enhanced chemiluminescence detection method (WesternBright ECL, Advasta, Menlo Park, CA, USA) using the ChemiDoc MP Imaging System (Bio-Rad), as previously described [49]. Quantification of the protein bands was performed using Image J Software version 1.8.0.

### 3.10. Statistical Analysis

The data were expressed as the mean ± SD from at least three independent experiments. Student’s t-test and one-way ANOVA analyses were performed with Past3 Software, which was used for statistical analysis of the data; differences between groups were considered statistically significant when *p* < 0.05.

## 4. Conclusions

The treatment of insulin-resistant human hepatic HepG2 and Hep3B cells with the combination of the phytochemicals NAR, HES, CUR, POL, and QRC (that mirror the nutraceutical formulation GliceFen^®^, Mivell, Fano, Italy) modulates the expression of several genes involved in the regulation of insulin resistance molecular mechanisms. In fact, the mixture of these five natural compounds increases the mRNA levels of the *INSR* gene, decreases the expression levels of *GLUT3* and *SEMA3E,* and, in only Hep3B cells, can induce an increase in the mRNA levels of the *SIRT1* gene. Notably, the inhibition of *SEMA3E* expression levels in HepG2 cells is synergistic, indicating that the combination of these five natural molecules can remarkably inhibit the pro-inflammatory mechanisms associated with T2DM pathology. In addition, our results show that the mixture of NAR, HES, CUR, POL, and QRC induces an increase in INSR protein levels and a decrease in semaphorin 3E levels in both the investigated in vitro models. Notably, the combination of these five bioactives decreased the activity of the pro-inflammatory enzyme caspase 1 and of the hyperglycemic enzyme DPPIV in the insulin resistant HepG2 and Hep3B cells. Noteworthy, the inhibition of DPPIV catalytic activity in Hep3B cells is synergistic, indicating that the mixture of these phytochemicals can counteract the establishment of a hyperglycemia condition and can inhibit the molecular pathways of insulin resistance that lead to T2DM development. In conclusion, our results indicate that the combination of NAR, HES, CUR, POL and QRC could be used as an innovative therapeutic strategy for the insulin resistance condition and could represent an effective innovative complementary approach to the T2DM pharmacological treatment.

## 5. Patents

Italian Patent Application: 102021000032909; Mivell, Fano, Italy.

## Figures and Tables

**Table 1:** Evaluation of the synergistic biological effects of the N + H and MIX treatments in HepG2 and Hep3B cells.

**HepG2**	***INSR*** **FOLD**	**Hep3B**	***INSR*** **FOLD**
INS + N	+2.60%	INS + N	+2.10%
INS + H	−4.30%	INS + H	−6.60%
INS + N + H	+36.90%	INS + N + H	+33.70%
**HepG2**	***SIRT1*** **FOLD**	**Hep3B**	***GLUT3*** **FOLD**
INS + N	+26.30%	INS + N	−7.90%
INS + H	+53.70%	INS + H	−11.90%
INS + N + H	+87.10%	INS + N + H	−38.40%
**HepG2**	***SEMA3E*** **FOLD**	**Hep3B**	**DPPIV Activity**
INS + N + H	−45.10%	INS + N + H	−16.30%
INS + C + P + Q	−43.40%	INS + C + P + Q	−15.60%
INS + MIX	−90.10%	INS + MIX	−35.50%

**Table 2:** Sequence of primers used for RTqPCR experiments.

Primer	Sequence
INSR F	TACCCTTCAAGAGATGATT
INSR R	CAGAAGAAGTGGTGAAGAC
GLUT2 F	TGGGCTGAGGAAGAGACTGT
GLUT2 R	AGAGACTGAAGGATGGCTCG
GLUT3 F	CAATGCTCCTGAGAAGATCATAA
GLUT3 R	AAAGCGGTTGACGAAGAGT
SIRT1 F	TAGGCGGCTTGATGGTAA
SIRT1 R	ATGGGTTCTTCTAAACTTGG
SEMA3E F	AAGTCAGATTCCATCACTGTGACAT
SEMA3E R	AGCAAAGTACTGTTGTTCTCTATGC
B2M F	GCCTGCCGTGTGAACCAT
B2M R	CATCTTCAAACCTCCATGATGCT

## Data Availability

Data is contained within the article or Supplementary Materials.

## Supplementary Materials

The following supporting information can be downloaded at: https://www.mdpi.com/article/10.3390/ijms24098071/s1.

## Abbreviations

Akt: Ak strain transforming; ASC, Apoptosis-associated speck-like protein containing a caspase-recruitment domain; B2M, β2-microglobulin; CUR, Curcumin; DPPIV, Dipeptidyl peptidase IV; FXR2, Fragile X mental retardation syndrome-related protein 2; GLP-1, Glucagon-like peptide 1; GLUT1, Glucose transporter type 1; GLUT3, Glucose transporter type 3; GLUT4, Glucose transporter type 4; HES, Hesperetin; HRP, Horseradish peroxidase; IL-1β, Interleukin-1β; IL-6, Interleukin-6; IL-18, Interleukin-18; INSR, Insulin Receptor; IRS, Insulin receptor substrate; mTORC2, mammalian Target of rapamycin complex 2; NAR, Naringenin; NLRP3, Nucleotide-binding domain, leucine-rich containing family, pyrin domain-containing-3; PBS, Phosphate Buffered Saline; PDK1, 3-phosphoinositide-dependent kinase 1; PI3K, Phosphoinositide 3-kinase; POL, Polydatin; QRC, Quercetin; RIP2, Receptor-interacting protein 2; ROS, Radical Oxygen Species; SDS-PAGE, SDS Polyacrylamide gel electrophoresis; SEMA3E, Semaphorin 3E; SIRT1, Sirtuin 1; T2DM, Type 2 diabetes mellitus; TBC1D4, TBC1 Domain Family member 4; TNF-α, Tumor Necrosis Factor-α.
