# Supplementary material for: The Combination of Natural Molecules Naringenin, Hesperetin, Curcumin, Polydatin and Quercetin Synergistically Decreases SEMA3E Expression Levels and DPPIV Activity in In Vitro Models of Insulin Resistance
Source: Int J Mol Sci. 2023 Apr 29;24(9):8071. doi: 10.3390/ijms24098071 (PMC10178687; doi:10.3390/ijms24098071)

**Supplementary Figure S1**: Uncropped blots used for the western blot images of Figure 4. Lanes indicated with § have been used for the preparation of Figure 4.

Figure 4A

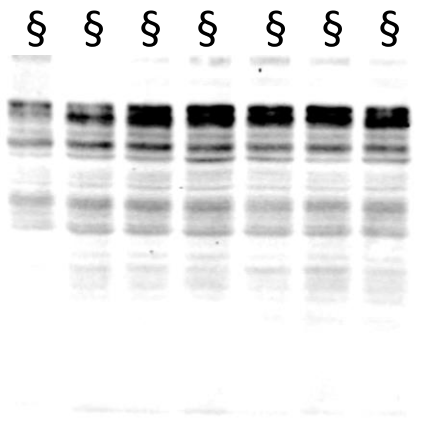


Figure 4D

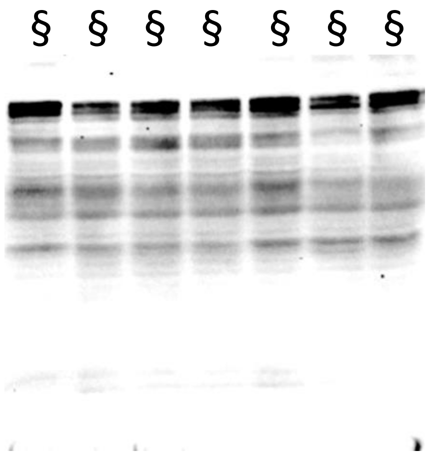


Figure 4A

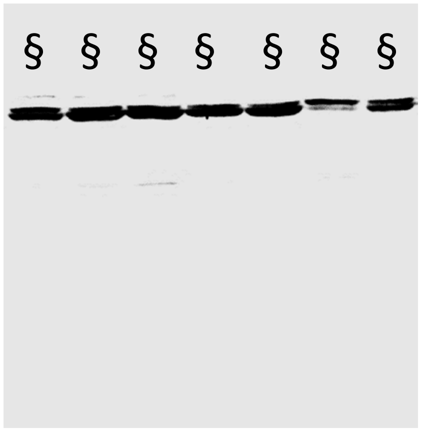


Figure 4D

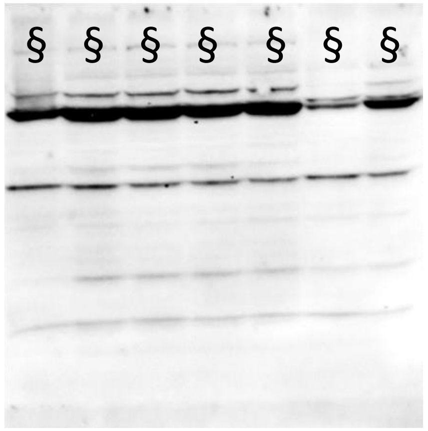


Figure 4A

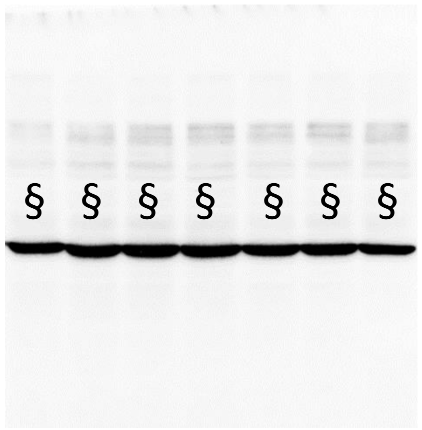


Figure 4D

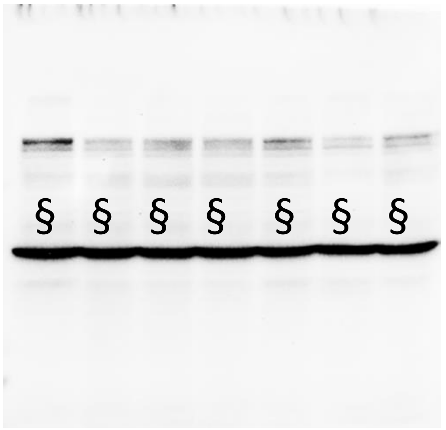

Supplement: Supplementary file 1 [file ijms-24-08071-s001.zip › ijms-2384194-supplementary.pdf]
